# Supplementary material for: Microclot array elastometry for integrated measurement of thrombus formation and clot biomechanics under fluid shear
Source: Nat Commun. 2019 May 3;10:2051. doi: 10.1038/s41467-019-10067-6 (PMC6499828; doi:10.1038/s41467-019-10067-6)
Supplement: Supplementary file 1 — Supplementary Information [file 41467_2019_10067_MOESM1_ESM.pdf]

Supplementary Information

Microclot array elastometry for integrated measurement of thrombus formation and clot biomechanics under fluid shear

Zhao et al.

Supplementary Figures

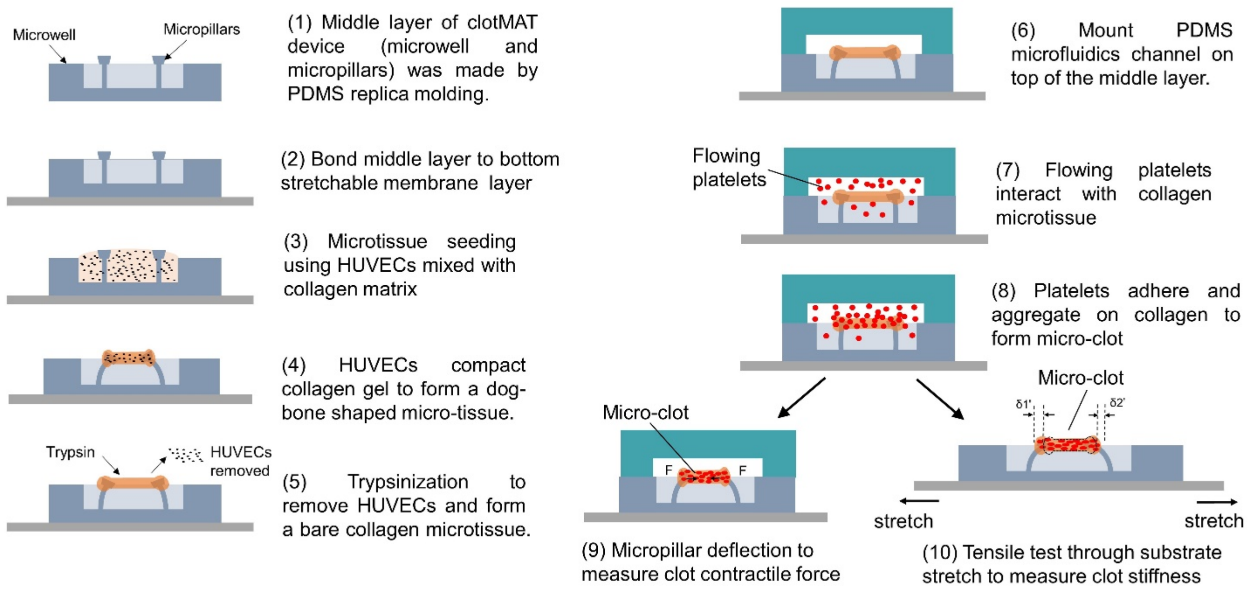

Supplementary Figure 1. Flow diagram shows the process of clotMAT device fabrication (steps 1-2), collagen microtissue preparation (steps 3-5), microclot formation (steps 6-8) and microclot mechanical property measurement (steps 9-10).

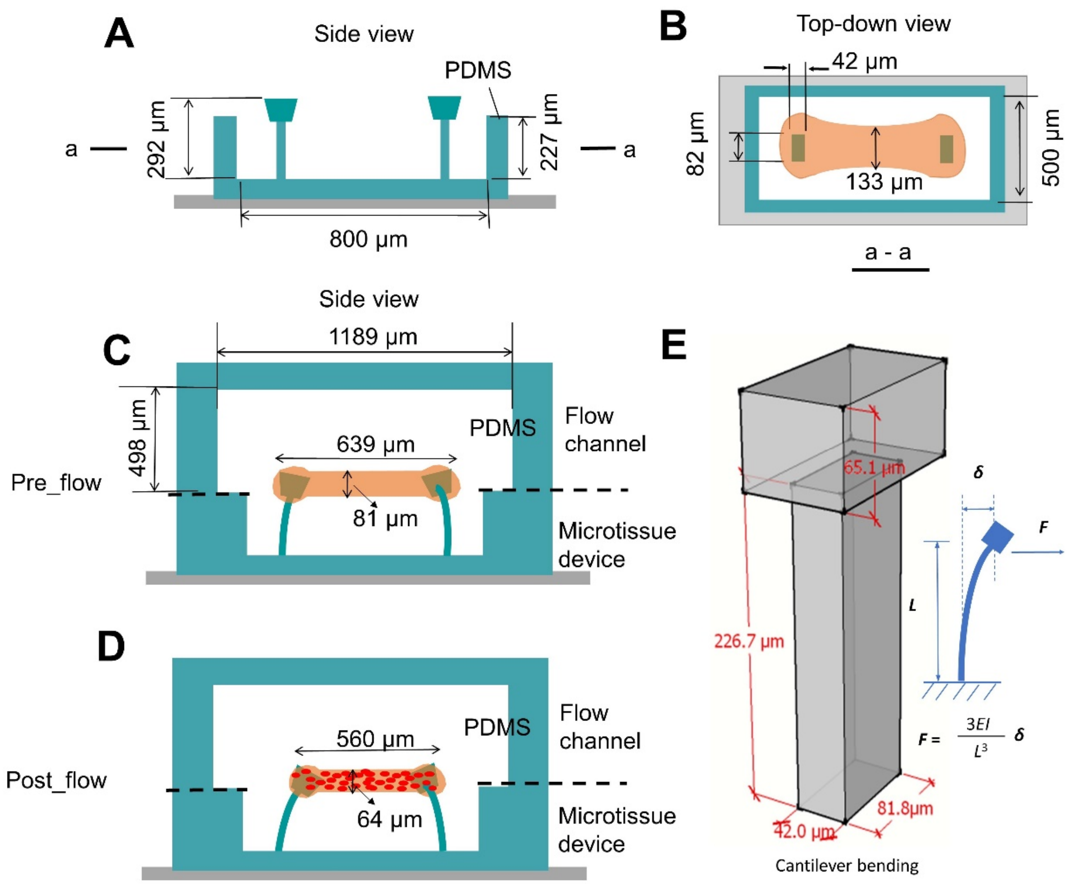

Supplementary Figure 2. Schematic side view (A) and top-down view (B) of the microwell and micropillars. Schematic side view shows the representative dimensions of the microtissue before flow experiment (pre_flow, C) and after flow induced platelet adhesion (post_flow, D). Flow channel is mounted on top of the microtissue array. (E) Three dimensional illustration of a single micropillar with a spring constant $3EI/L^3$ =120 nN µm^{-1}.

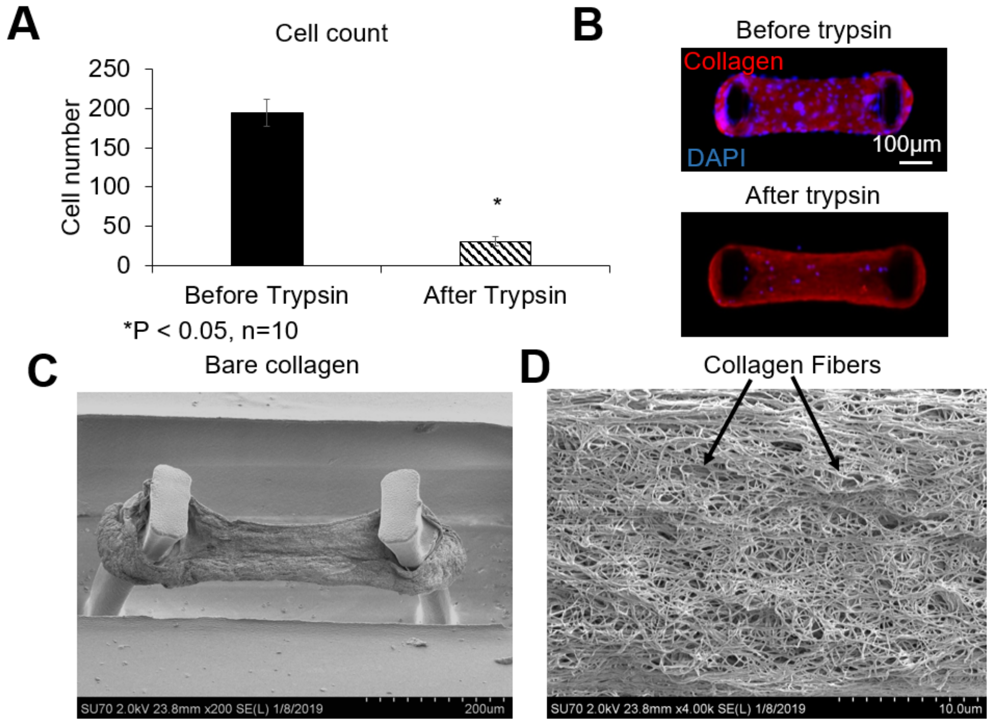

E

	Width (µm)	Length (µm)	Area (10^3 µm^2)	Thickness (µm)
Bare collagen	132.7 ± 17.2	638.9 ± 21.3	96.7 ± 10.6	80.7 ± 5.9

Supplementary Figure 3. The formation of bare collagen microtissue through trypsinization of HUVEC-populated microtissue. Cell count (A) and immunofluorescence staining images (B) showed that almost all HUVECs were removed from the microtissue through trypsin treatment, leaving behind a bare collagen matrix. (C) SEM image of a bare collagen microtissue hanging between a pair of micropillars after trypsinization. (D) Enlarged view of a portion of the bare collagen matrix showing collagen fiber meshwork. (E) Dimensions of the bare collagen microtissues. Sample size: 113 for width, 25 for length, 108 for area and 16 for thickness. Data are presented as mean ± standard deviation.

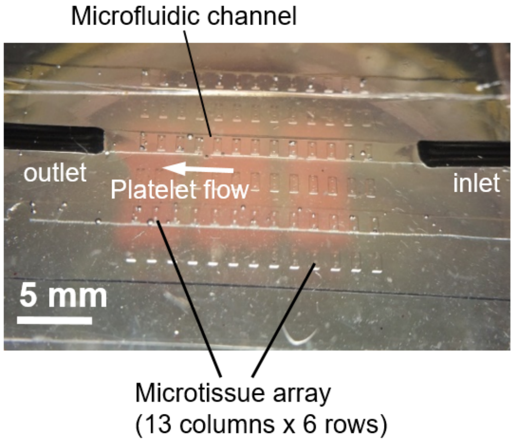

Supplementary Figure 4. Actual setup of the clotMAT device. A region of the microtissue array is shown. The microfluidic channels in the top PDMS layer was aligned with individual rows of collagen microtissues in the middle layer.

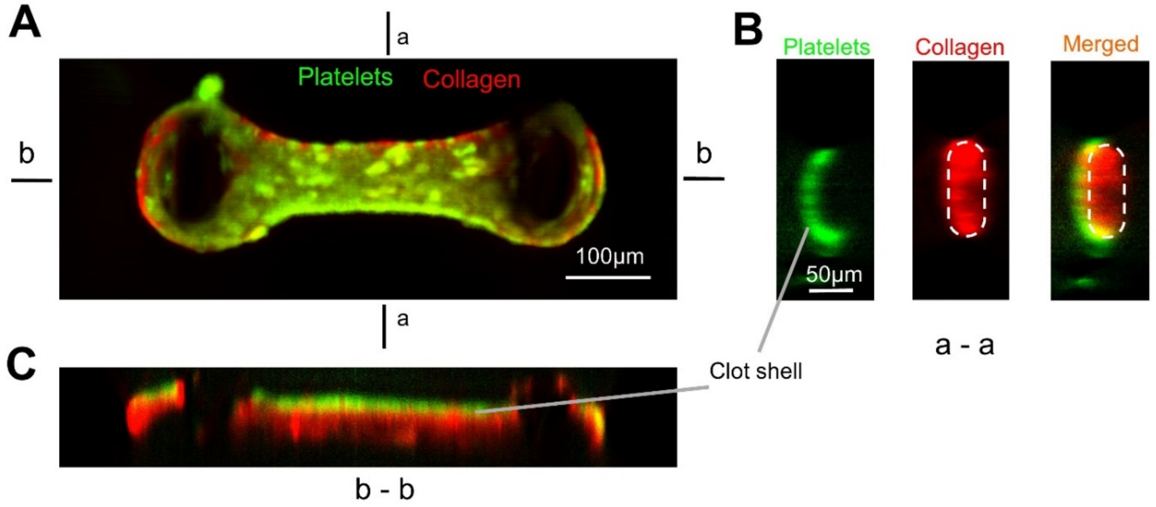

D

	Width (μm)	Length (μm)	Area (x10^3 μm^2)	Thickness (μm)
Microclot	83.3 ± 9.2	559.8 ± 19.0	63.0 ± 6.6	64.1 ± 5.8

E

	Collagen before flow	Whole clot	Clot shell
Thickness (μm)	80.7 ± 5.9	64.1 ± 5.8	28.2 ± 7.6

Supplementary Figure 5. Confocal fluorescent top-down view (A), cross-sectional view (B) and side view (C) of a microclot formed using citrated healthy PRP. Platelets are labeled in green and collagen is labeled in red. In (B) and (C), it can be seen that a thick clot shell (green) covers the bare collagen core (red). (D) The dimensions of formed microclots. (E) Comparison of the layer thickness between bare collagen microtissue before platelet flow (no compaction), a whole microclot and the clot shell in the microclot. It can be seen that the thickness of the whole microclot is smaller than that of the bare collagen due to platelet contraction-mediated tissue compaction. n = 14 for microclot dimension measurement. Data are presented as mean ± standard deviation.

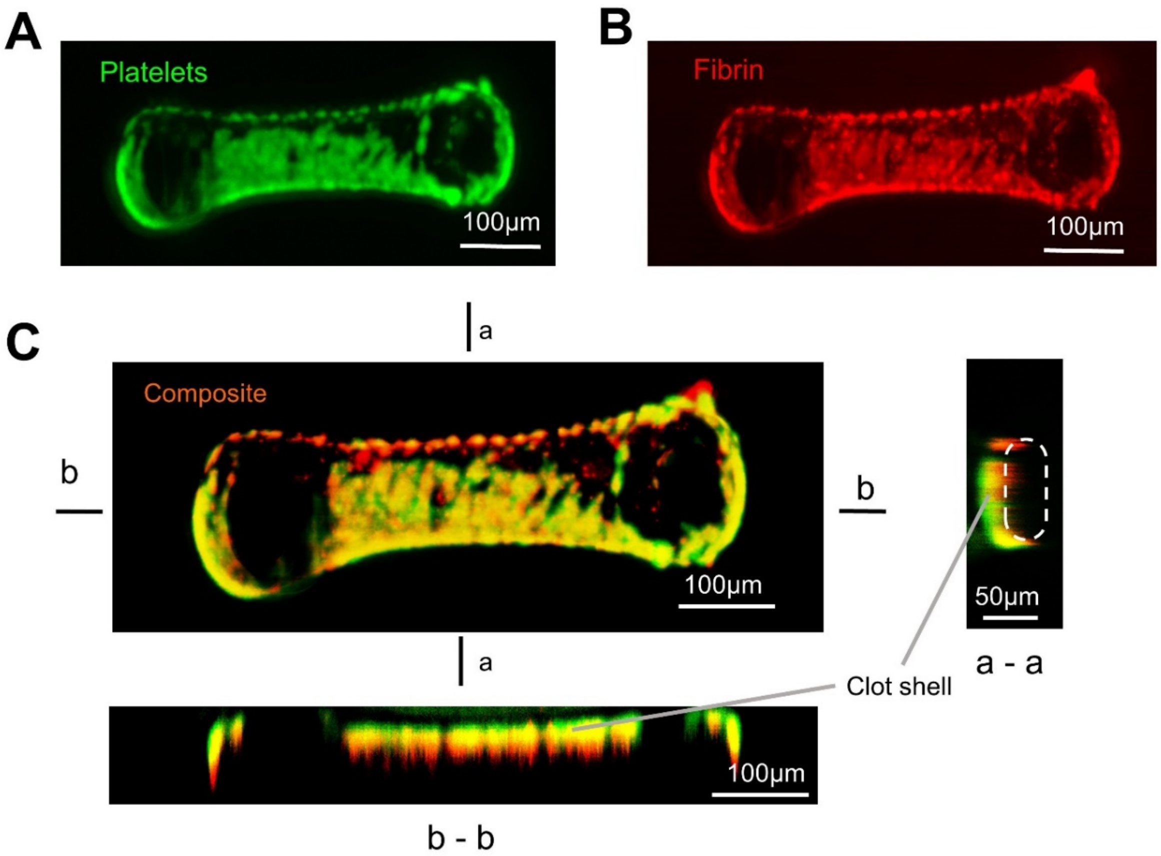

Supplementary Figure 6. Confocal fluorescent top-down view of platelets (green, A) and fibrin (red, B) in a microclot. Fibrin was formed during flow experiment at 500 s^{-1} shear rate for 30 minutes using citrated healthy PRP containing Alexa Fluor 594-conjugated fibrinogen (Fb-594) at 1:20 mixing ratio (Fb-594 : PRP in volume). (C) Confocal fluorescent top-down view, cross-sectional view (a-a) and side view (b-b) of the same microclot. Platelets and fibrin signal channels were merged. It can be seen that platelets and fibrin co-localize in the clot shell.

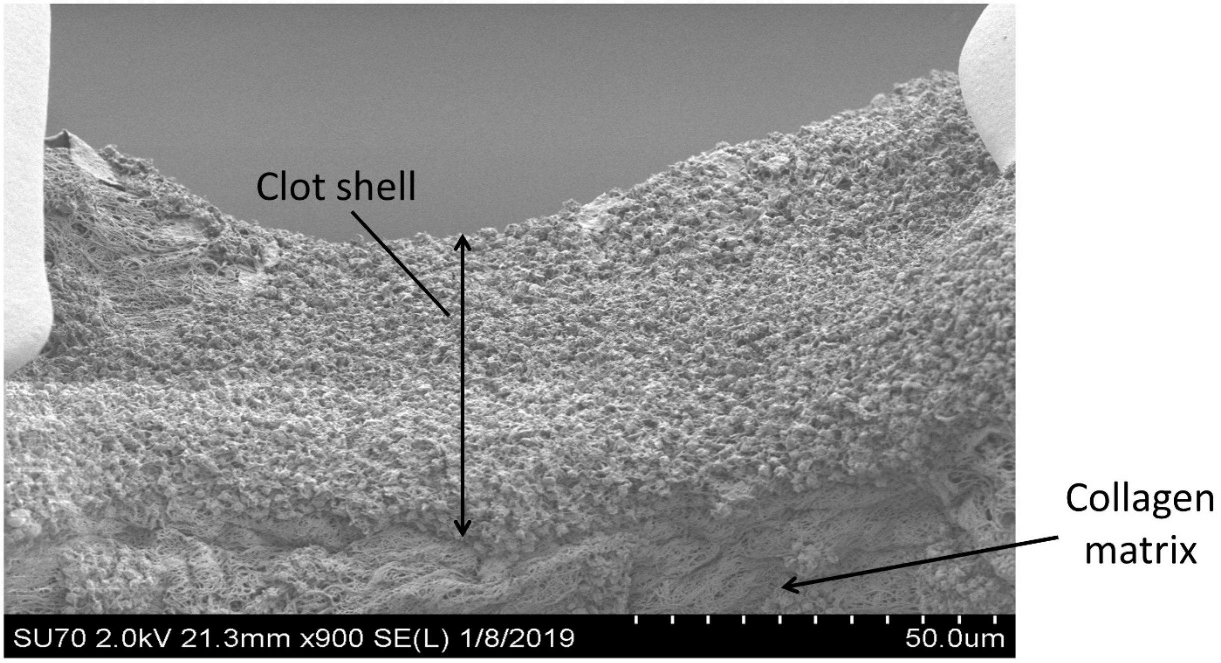

Supplementary Figure 7. SEM image showing a representative microclot (portion between the micropillars). It can be seen that majority of the surface is covered by the clot shell. The region close to the edge of the tissue (bottom of the image) shows the interface between the clot shell and collagen matrix. Note the thickness of the clot shell is reduced as compared to fresh sample due to the shrinkage caused by sample preparation for SEM imaging.

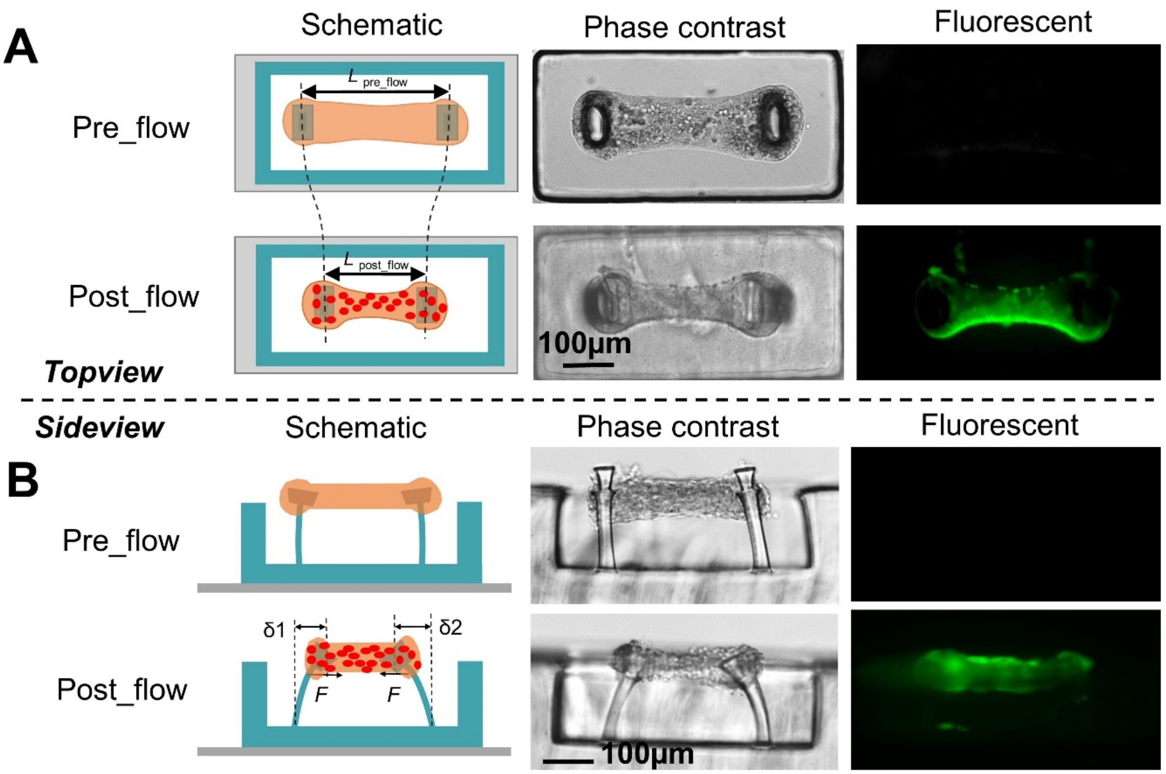

Supplementary Figure 8. Schematic, phase contrast and fluorescence topview (A) and sideview (B) images of a microtissue before and after flow induced platelet adhesion. Micropillar deflection (δ1 and δ2) can be easily detected in post-flow conditions, which was used to calculate microclot generated contraction force according to cantilever bending theory.

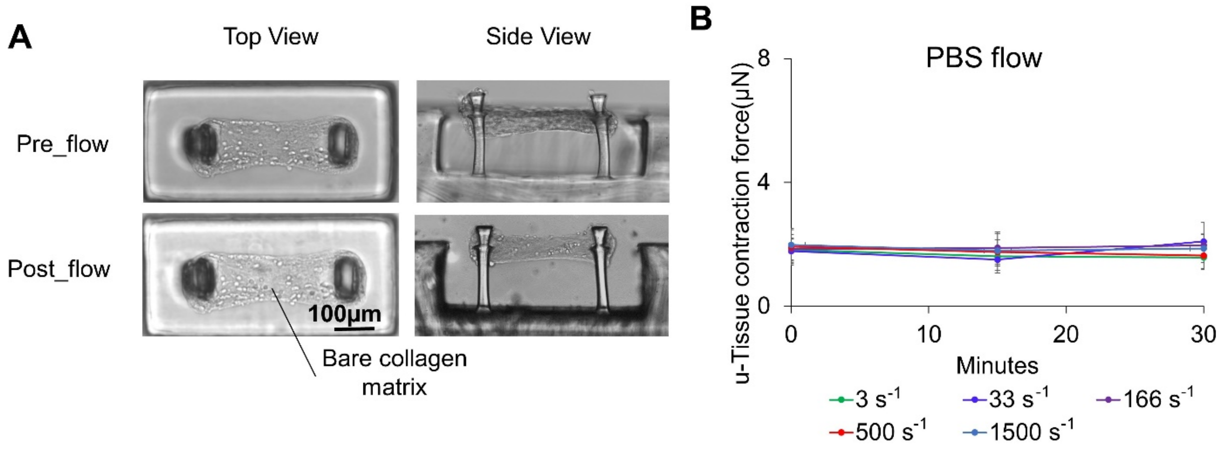

Supplementary Figure 9. PBS flow, in the absence of platelets, had no effect on bare collagen microtissue. There was no obvious change in either microtissue morphology (A) or contractile force (B) after 30 mins of PBS flow. Data are mean ± standard deviation.

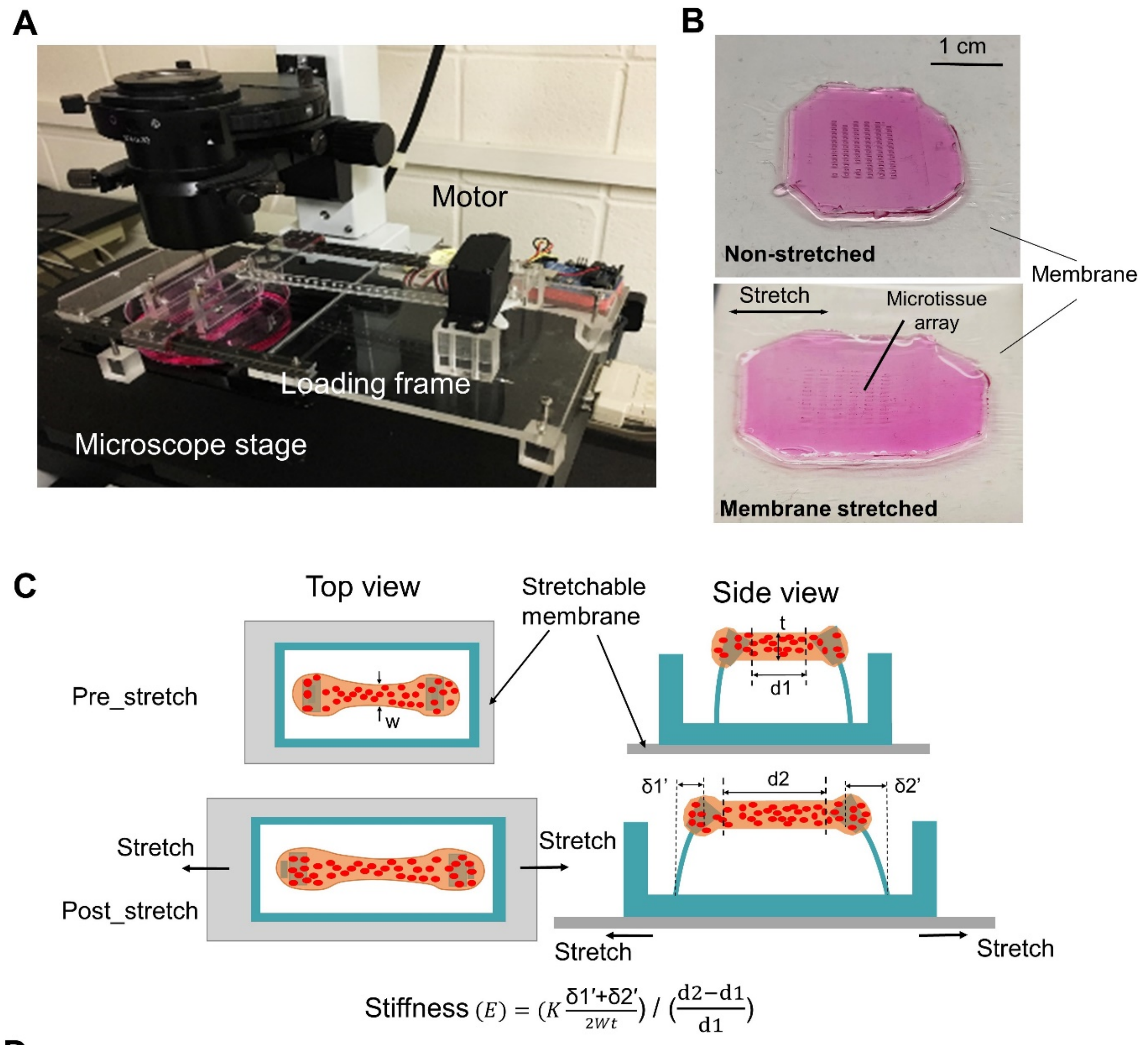

D

K	K(δ1′+δ2′)/2	W	t	Stress	Strain	Stiffness
0.12 μN μm^{-1}	10.29 μN	84.67 μm	65.1 μm	1.87 kPa	10.10%	18.49 kPa

Supplementary Figure 10. Mechanical stretching system setup. (A) clotMAT device was mounted on a motor driven loading frame that is situated on top of the microscope stage. (B) Microtissue array before and after stretching through the bottom membrane. (C) Schematic of top view and side view of a microtissue before and after membrane stretch. (D) A set of representative microclot stiffness measurement data.

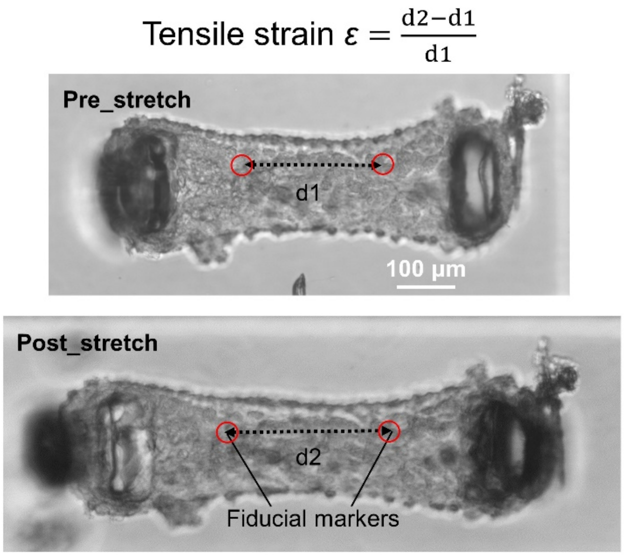

Supplementary Figure 11. Calculation of stretching-induced tensile strain in the microtissue. The distances between two fiducial markers before and after tensile test were determined through image analysis and the tensile strain was calculated as $\varepsilon = (d2 - d1)/d1$. Only the belly region of the microtissue was used in strain analysis to match with the region where cross-sectional area was measured.

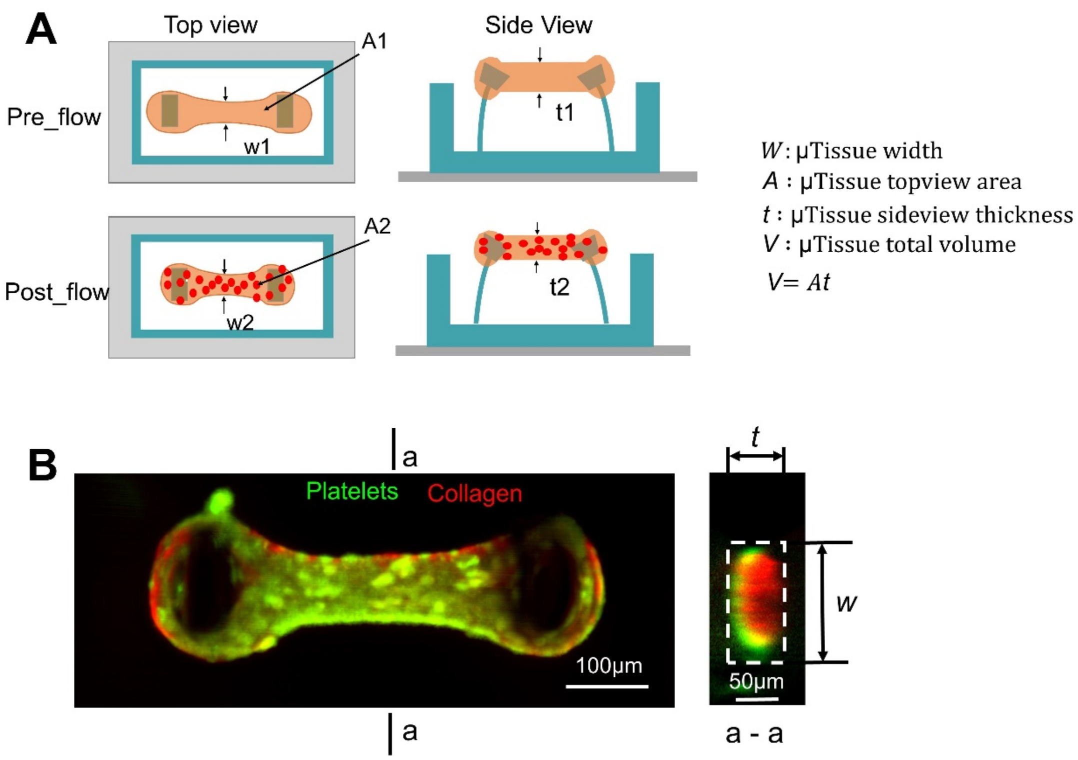

Supplementary Figure 12.Confocal imaging to determine microtissue geometrical parameters. (A) Schematic shows microtissue dimensional parameters. (B) Confocal fluorescent topview and cross-sectional view (a-a) images of a microclot. Cross-sectional view was constructed from confocal stacks and was used to determine the microtissue thickness t. Microtissue width (w) can be determined through microtissue topview using conventional microscopy.

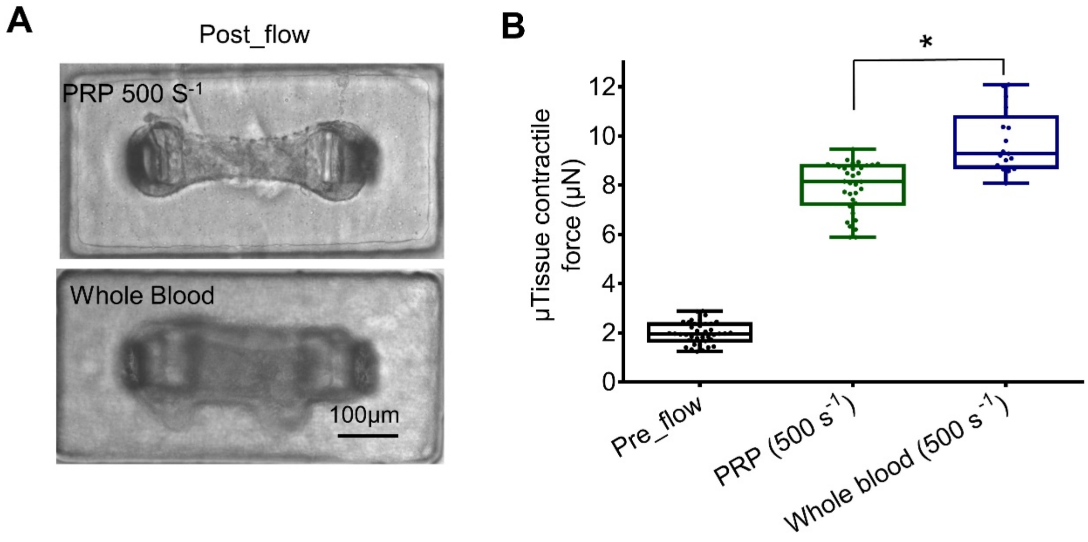

Supplementary Figure 13. Whole blood flow caused the formation of bigger microclots that generate higher contractile force as compared to PRP flow. (A) Bright field images of microclots formed under PRP flow and whole blood flow. (B) Contractile force measurement of microclots formed under PRP flow and whole blood flow. *$P < 0.05$, $n > 10$, each dot in box plot represents an independent measurement.

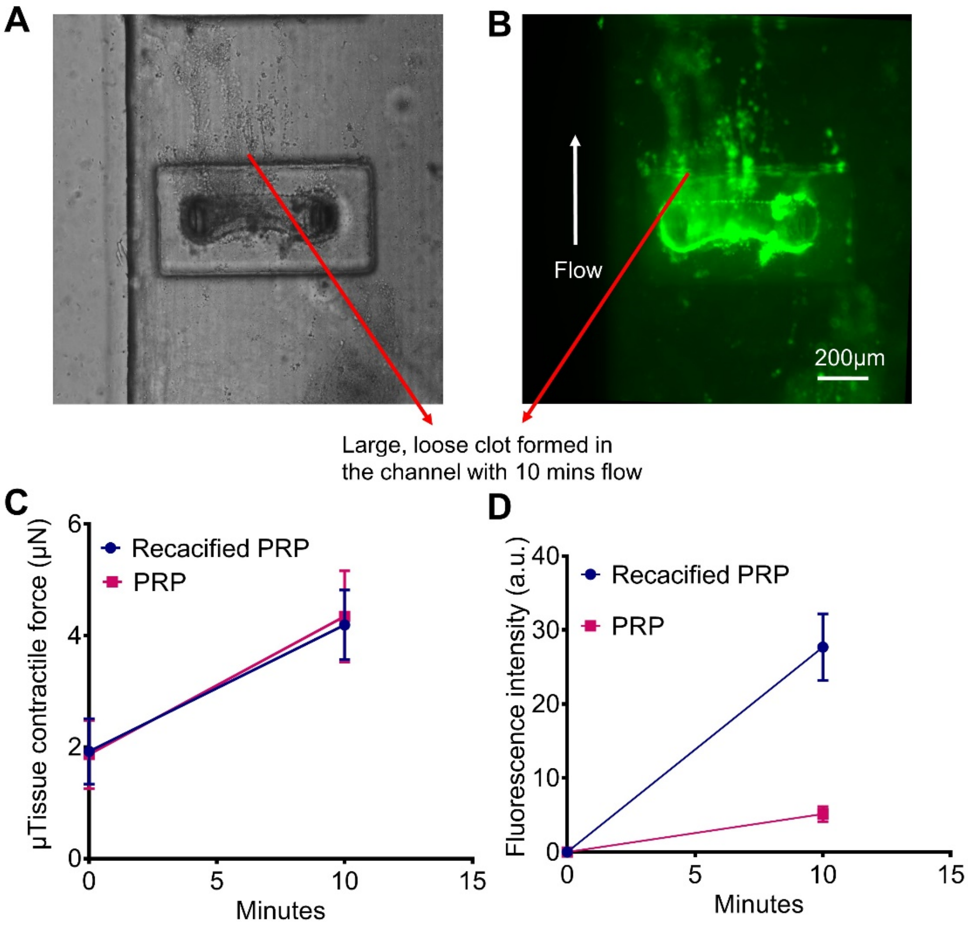

Supplementary Figure 14. Recalcified PRP flow caused the formation of large, loose clots in the channel. Bright field (A) and fluorescent (B) images of clot structure after 10 mins of recalcified PRP flow. The massive, loose clot blocked the channel after 10 mins of flow. Contractile force (C) and fluorescence intensity (D) measurement for microclots formed under normal PRP flow and recalcified PRP flow. Both direction of the error value equal to the standard deviation for all figures. Data are mean ± standard deviation.

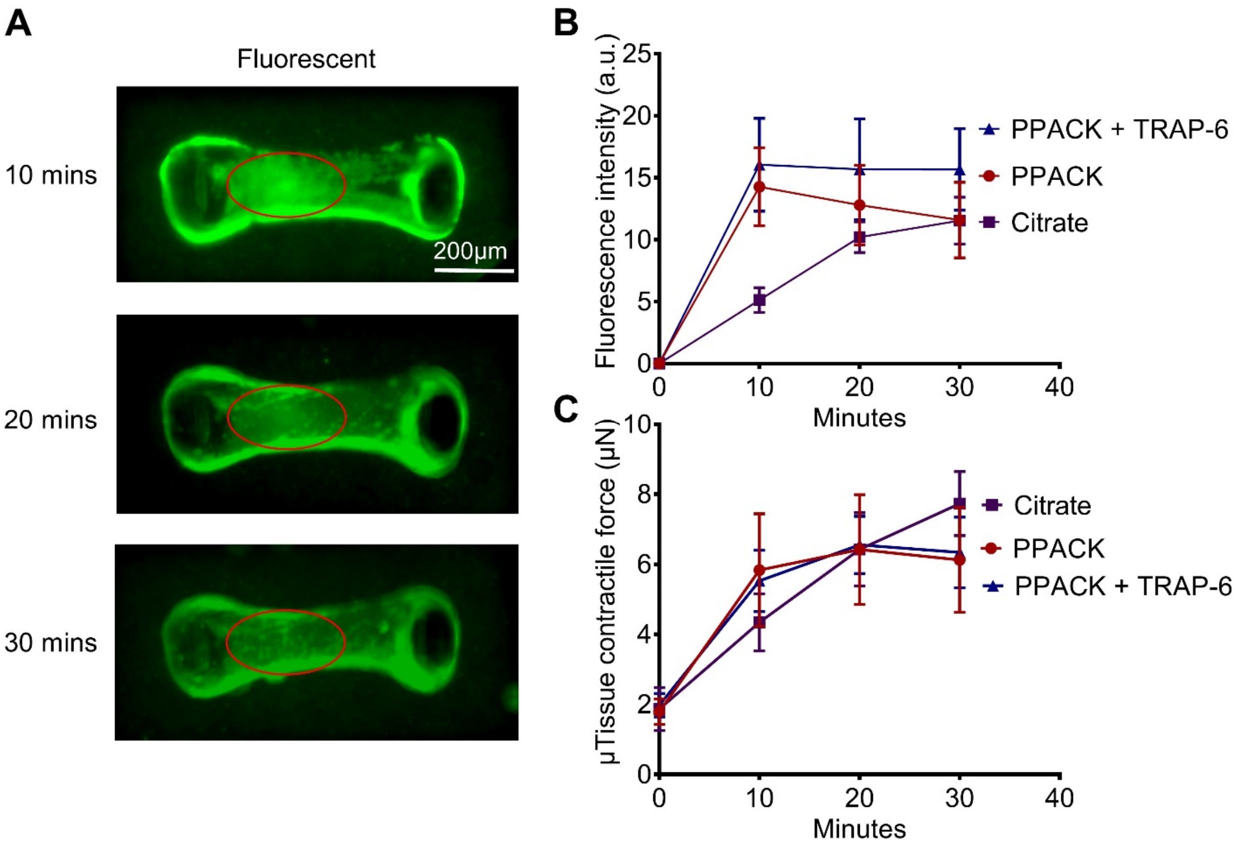

Supplementary Figure 15. PRP flow experiment using PPACK as anticoagulant. (A) Fluorescent images of a representative microclot during 30 mins of PPACK-PRP flow. It can be seen that a large size clot formed on the microtissue surface by 10 mins (indicated by red circle), but it disappeared at 20 and 30 mins, suggesting platelet detachment and thromboembolism under shear flow. Fluorescence intensity (B) and contractile force (C) measurements of microclots formed under normal citrated PRP flow, PPACK-PRP flow and PPACK-PRP flow treated with TRAP-6. Compared to the citrated group, PPACK group showed more platelet binding and higher contractile force generation in the early stage (first 10 mins) of the flow experiment. Fluorescence intensity plateaued and then decreased and contractile force also decreased in the mid to late stage (10 – 30 mins) of the flow experiment for PPACK group, likely due to platelet thromboembolism. Treatment of the PPACK group with thrombin receptor activator peptide 6 (TRAP 6) rescued the decrease in fluorescence intensity during mid to late stage of the flow experiment but had no effect on contractile force. Data are mean ± standard deviation.

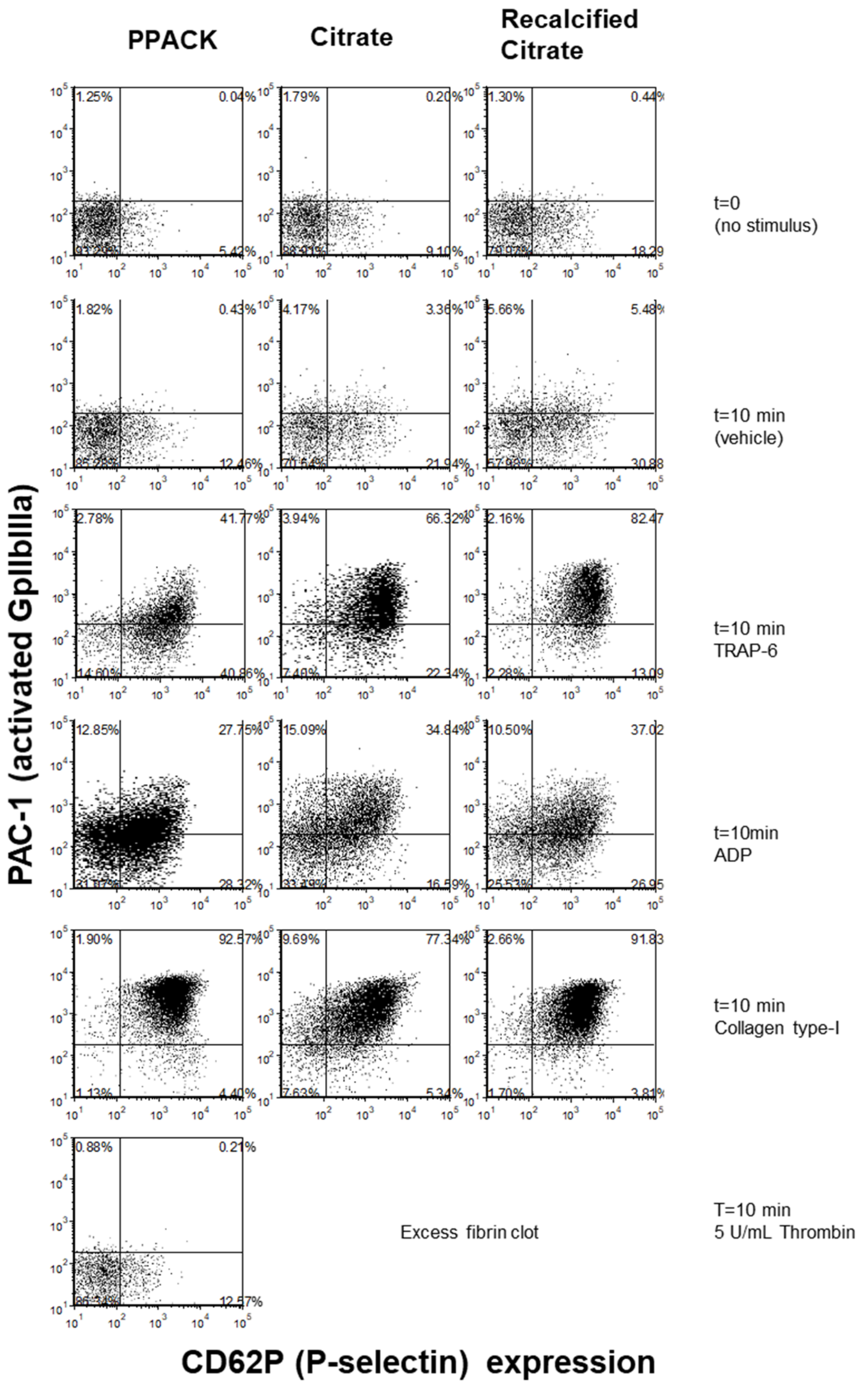

Supplementary Figure 16. Platelet activation studies. PRP prepared in different anti-coagulants was subjected to platelet agonists. Dot plot demonstrating P-selectin (CD62P) and activated GpIIbIIIa (mAb PAC-1) differentially increased by different stimuli. TRAP-6 triggered the highest activation, 82.47% of platelets in recalcified citrate. Thrombin resulted in extensive fibrin formation in PRP in the case of citrated samples and thus platelet events were absent due to blood clotting in the bottom panel. Thrombin action was not observed when PPACK was anti-coagulant, since PPACK inhibits thrombin mediated platelet activation.

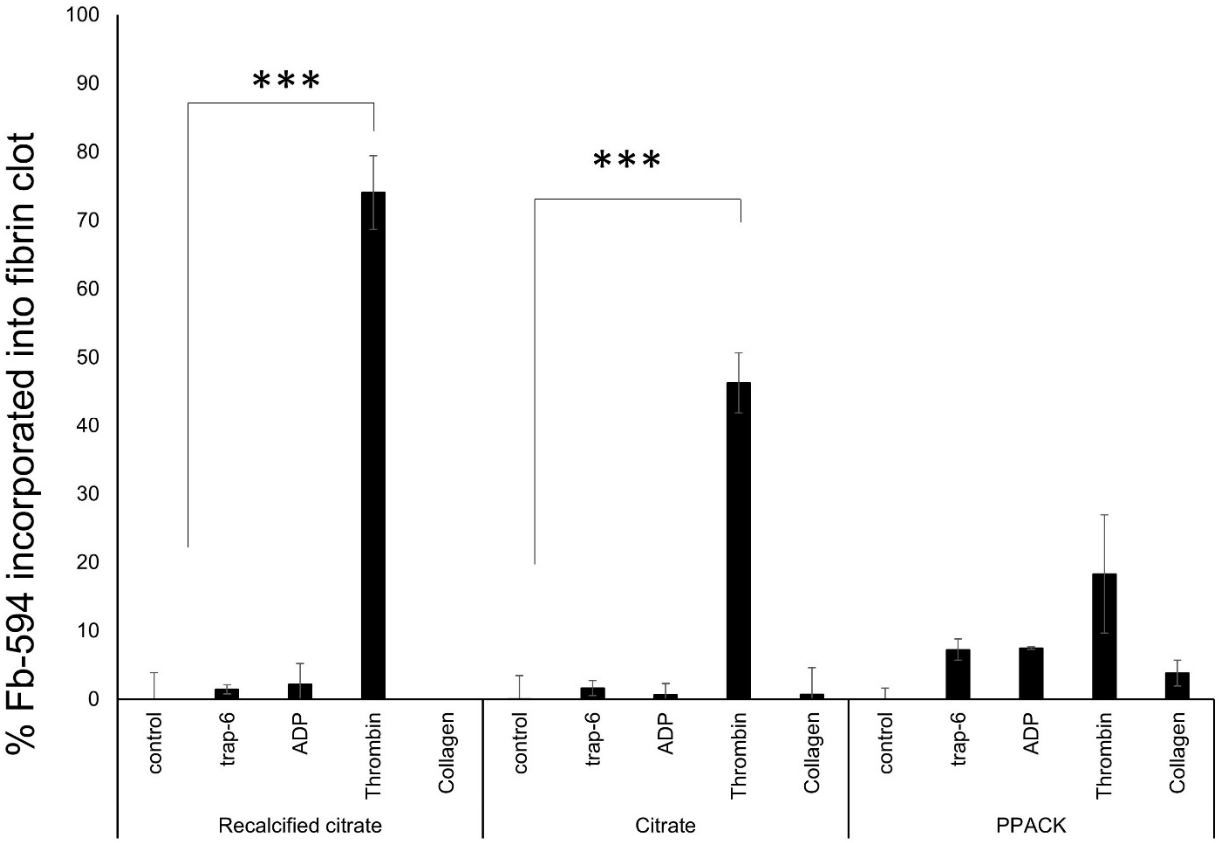

Supplementary Figure 17. Fibrin formation under static conditions. Fibrin formation is most prominent when using thrombin, but it is partially inhibited when using PPACK as anti-coagulant. Data are mean ± S.D. ***P<0.001.

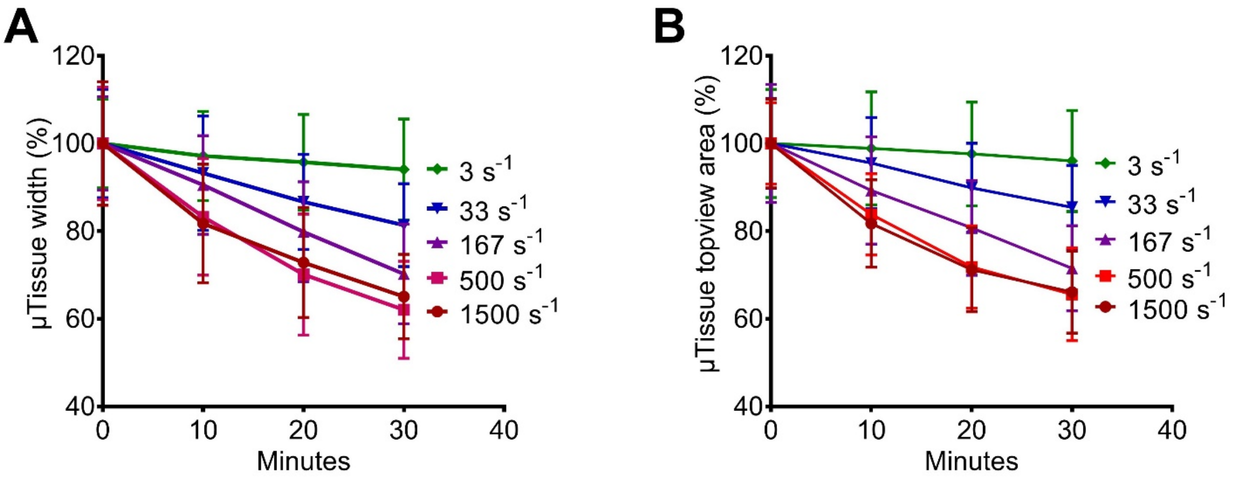

Supplementary Figure 18. Evolution of microtissue geometrical parameters under different shear flow conditions. (A) Change of microtissue width (w) over a 30 mins time period for different flow rates. (B) Change of microtissue topview area over a 30 mins time period for different flow rates. Experiments for each flow rate were performed with sufficient repetition in independent microtissue devices. Data are mean ± standard deviation.

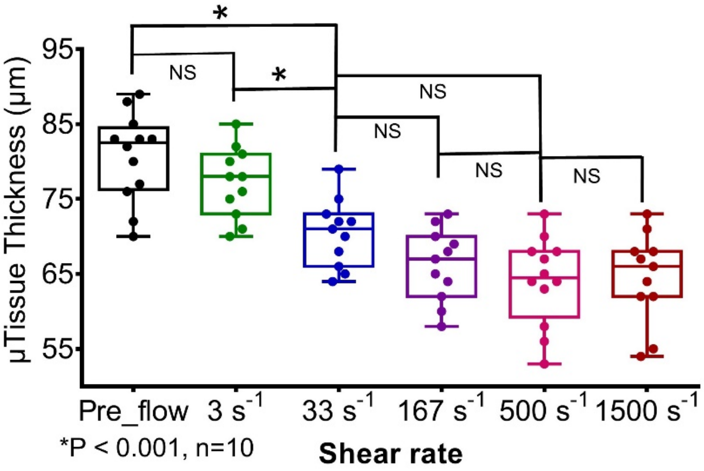

Supplementary Figure 19. Microtissue thickness (t) measured after 30 mins of platelet flow under different shear flow conditions. Experiments for each flow rate were performed with sufficient repetition in independent microtissue devices. *P < 0.05 compared with pre_flow based on ANOVA, n > 8, each dot in box plot represents an independent experiment.

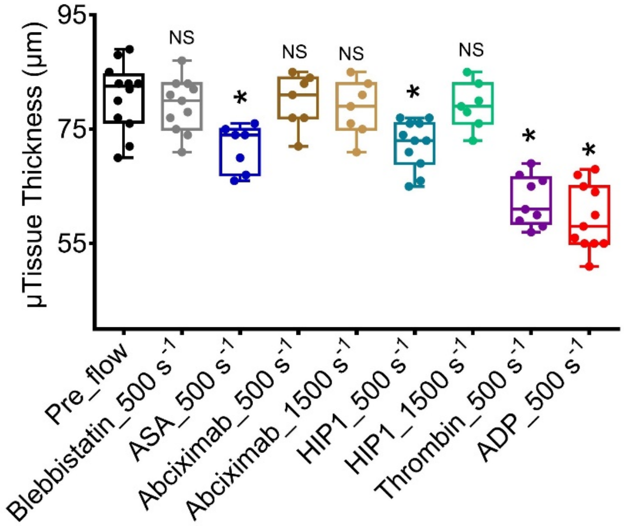

Supplementary Figure 20. Microtissue thickness (t) measured after 30 mins of platelet flow under different biochemical treatments. Experiments for each flow condition were performed with sufficient repetition in independent microtissue devices. *P < 0.05 compared with pre_flow based on ANOVA, n > 8, each dot in box plot represents an independent experiment. The number associated with each treatment represents the local shear rate (either 500 s^{-1} or 1500 s^{-1}).

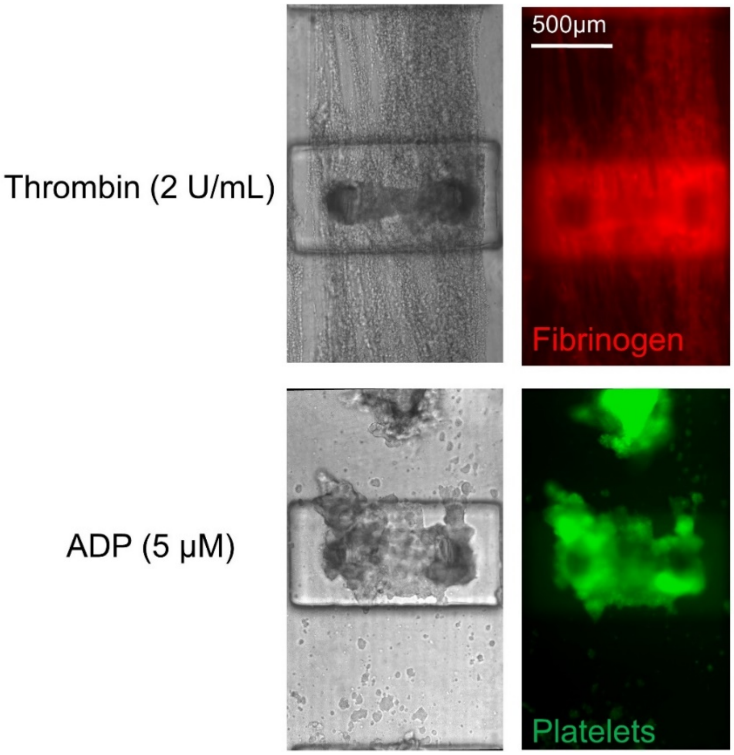

Supplementary Figure 21. Citrated PRP flow under thrombin and ADP treatment. Alexa fluor-594 conjugated fibrinogen was mixed with citrated PRP and the mixture was treated with thrombin (2 U/ml, top panels) or ADP (5 µM, bottom panels) under flow. Large amount of fibrin formation (red) blocked the flow channel after 10 minutes of flow in the presence of thrombin (top panels). ADP caused large platelet-rich clot formation (green), but the clots did not block the flow channel.

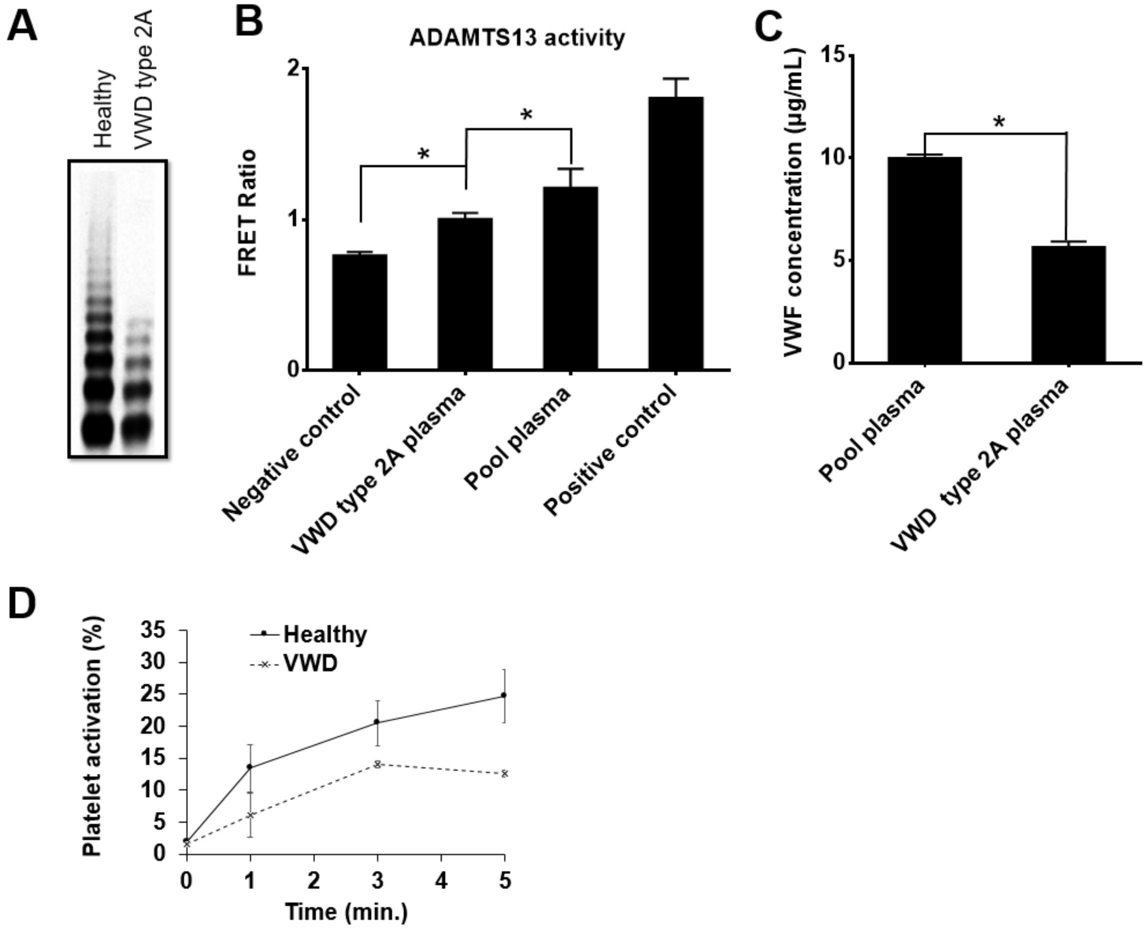

Supplementary Figure 22. Characterization of the VWD Type 2A patient sample. (A) Comparison of western-blot result of VWF multimer distribution for patient plasma (VWF Type 2A) vs. healthy normal control plasma. Patient plasma lacks high molecular mass VWF multimers. (B) ADAMTS-13 activity measured using XS-VWF FRET substrate. Data are presented for 30 min. Negative control: no plasma; Positive control: 1U recombinant ADAMTS13/mL; Pool plasma: Normal pooled plasma from 5 healthy, adult human volunteers. * $P<0.05$ with respect to negative control based on ANOVA and Tukey post-test. (C) VWF concentration determined by a flow cytometer-bead sandwich assay. Patient plasma has lower VWF concentration as compare to health donor sample, which is expected for VWD Type 2A patients. (D) In shear induced platelet activation (SIPAct) assays, VWD Type 2A plasma (VWD) displayed reduced platelet activation compared to healthy control. Data are mean ± standard deviation.

A

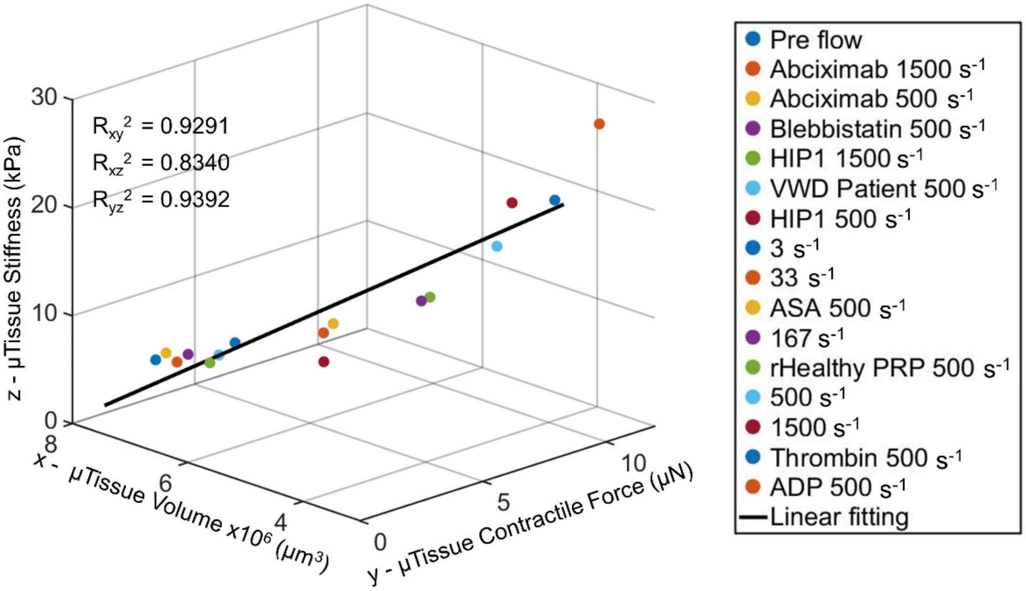

B

	Contractile force (μN)		Stiffness (kPa)		Volume (10^6 μm^3)	
	Mean	STDEV	Mean	STDEV	Mean	STDEV
Pre_flow	1.86	0.56	5.74	0.84	7.34	0.50
Abciximab 1500 s^{-1}	2.13	0.42	5.79	0.95	7.09	0.46
Abciximab 500 s^{-1}	2.15	0.45	6.26	1.29	7.29	0.59
Blebbistatin 500 s^{-1}	2.17	0.43	6.80	1.00	6.91	0.45
HIP1 1500 s^{-1}	2.45	0.44	6.28	1.06	6.65	0.56
VWD Patient 500 s^{-1}	2.84	0.77	6.69	0.93	6.66	0.59
HIP1 500 s^{-1}	3.57	0.86	8.26	1.45	5.15	0.32
3 s^{-1}	3.29	1.17	7.65	1.35	6.57	0.51
33 s^{-1}	4.50	0.80	9.52	1.35	5.55	0.36
ASA 500 s^{-1}	5.22	0.83	9.61	1.93	5.69	0.69
167 s^{-1}	6.24	1.04	12.94	1.72	4.60	0.41
rHealthy PRP 500 s^{-1}	6.85	1.07	12.63	1.86	4.71	0.36
500 s^{-1}	7.81	0.98	18.01	2.79	3.96	0.25
1500 s^{-1}	8.54	0.90	21.42	2.88	4.00	0.34
Thrombin 500 s^{-1}	9.41	0.90	21.69	3.12	3.64	0.20
ADP 500 s^{-1}	10.60	1.12	28.36	6.13	3.37	0.28

Supplementary Figure 23. (A) The correlation between microclot mechanical properties and the level of microclot retraction. Strong correlation between microclot stiffness and microclot contractile force (R_{yz}^2), between microclot contractile force and microclot volume (R_{xy}^2) and between microclot stiffness and microclot volume (R_{xz}^2) is observed. (B) Table lists the raw data used to construct the correlation plot.
