# Supplementary material for: Microclot array elastometry for integrated measurement of thrombus formation and clot biomechanics under fluid shear
Source: Nat Commun. 2019 May 3;10:2051. doi: 10.1038/s41467-019-10067-6 (PMC6499828; doi:10.1038/s41467-019-10067-6)
Supplement: Supplementary file 3 — Description of Additional Supplementary Files [file 41467_2019_10067_MOESM3_ESM.pdf]

Description of Additional Supplementary Files

File name: Supplementary Movie 1

Description: Time-lapsed movie shows microtissue formation process. A collagen gel pre-seeded in the microwell was compacted into a dog-bone shaped microtissue by the collective contraction of cells. Microtissue forms within 18 hours after seeding. Endothelial cells trapped in the collagen matrix were then removed through trypsinization (not shown in this movie), leaving a suspended, bare collagen matrix for recruiting platelets under shear flow.

File name: Supplementary Movie 2

Description: Dynamic formation of a microclot under platelet flow over a 4 min time period. Platelets were fluorescently labeled. The gradual increase in measured fluorescence intensity indicates increasing platelet accumulation and aggregation on the bare collagen microtissue. Note for demonstration purpose, 10 μM ADP was added in the flowing PRP to speed up platelet adhesion and microclot formation.

File name: Supplementary Movie 3

Description: Finite element simulated microclot retraction process. The active contraction of the platelet population was represented by the contractility of every single elements whose constitutive material consist of a neo-Hookean solid component and an isotropic contractile stress component. Due to the geometric restriction by two micropillars at both ends, microclot contraction causes volume shrinkage and development of necking in the middle section, mimicking the microclot retraction process observed in the experiment. Simulated effective stress contour was plotted over deformed model geometry.
