# Supplementary material for: Microclot array elastometry for integrated measurement of thrombus formation and clot biomechanics under fluid shear
Source: Nat Commun. 2019 May 3;10:2051. doi: 10.1038/s41467-019-10067-6 (PMC6499828; doi:10.1038/s41467-019-10067-6)
Supplement: Supplementary file 7 — Reporting Summary [file 41467_2019_10067_MOESM7_ESM.pdf]

nature research

Corresponding author(s): Ruogang Zhao, Sriram Neelamegham

Reporting Summary

Nature Research wishes to improve the reproducibility of the work that we publish. This form provides structure for consistency and transparency in reporting. For further information on Nature Research policies, see Authors & Referees and the Editorial Policy Checklist.

Statistical parameters

When statistical analyses are reported, confirm that the following items are present in the relevant location (e.g. figure legend, table legend, main text, or Methods section).

n/a	Confirmed	
☐	☒	The exact sample size (*n*) for each experimental group/condition, given as a discrete number and unit of measurement
☐	☒	An indication of whether measurements were taken from distinct samples or whether the same sample was measured repeatedly
☐	☒	The statistical test(s) used AND whether they are one- or two-sided<br>*Only common tests should be described solely by name; describe more complex techniques in the Methods section.*
☐	☒	A description of all covariates tested
☐	☒	A description of any assumptions or corrections, such as tests of normality and adjustment for multiple comparisons
☐	☒	A full description of the statistics including central tendency (e.g. means) or other basic estimates (e.g. regression coefficient) AND variation (e.g. standard deviation) or associated estimates of uncertainty (e.g. confidence intervals)
☒	☐	For null hypothesis testing, the test statistic (e.g. *F*, *t*, *r*) with confidence intervals, effect sizes, degrees of freedom and *P* value noted<br>*Give P values as exact values whenever suitable.*
☒	☐	For Bayesian analysis, information on the choice of priors and Markov chain Monte Carlo settings
☒	☐	For hierarchical and complex designs, identification of the appropriate level for tests and full reporting of outcomes
☐	☒	Estimates of effect sizes (e.g. Cohen's *d*, Pearson's *r*), indicating how they were calculated
☐	☒	Clearly defined error bars<br>*State explicitly what error bars represent (e.g. SD, SE, CI)*

Our web collection on statistics for biologists may be useful.

Software and code

Policy information about availability of computer code

Data collection	HCImageLive was used to capture bright field and fluorescent images
Data analysis	GraphPad Prism 6 was used to perform statistical analysis. ImageJ 1.51s was used to analyze the fluorescence intensity.

For manuscripts utilizing custom algorithms or software that are central to the research but not yet described in published literature, software must be made available to editors/reviewers upon request. We strongly encourage code deposition in a community repository (e.g. GitHub). See the Nature Research guidelines for submitting code & software for further information.

Data

Policy information about availability of data

All manuscripts must include a data availability statement. This statement should provide the following information, where applicable:

- Accession codes, unique identifiers, or web links for publicly available datasets
- A list of figures that have associated raw data
- A description of any restrictions on data availability

All data supporting the findings of this study are available within the article and its supplementary information files or from the corresponding author upon reasonable request.

Field-specific reporting

Please select the best fit for your research. If you are not sure, read the appropriate sections before making your selection.

☒ Life sciences ☐ Behavioural & social sciences ☐ Ecological, evolutionary & environmental sciences

For a reference copy of the document with all sections, see nature.com/authors/policies/ReportingSummary-flat.pdf

Life sciences study design

All studies must disclose on these points even when the disclosure is negative.

Sample size	For measurement of microtissue mechanical properties, geometries and fluorescent intensity, at least 3 independent groups of blood samples and 10 representative microtissues were used for quantification. For measurement of each coagulation treatment condition, at least 3 independent groups of blood samples and 10 representative microtissues were used for quantification. For measurement of patient plasma sample, three independent flow experiments were conducted for analysis. Sample size was chosen based on the common practice in the field.
Data exclusions	No data was excluded from the analysis.
Replication	For measurement of microtissue mechanical properties, geometries and fluorescent intensity, at least 3 replications using independent blood samples were performed. For measurement of patient plasma sample, three independent flow experiments were replicated.
Randomization	not applicable
Blinding	not applicable

Reporting for specific materials, systems and methods

Materials & experimental systems

n/a	Involved in the study	
☒	☐	Unique biological materials
☐	☒	Antibodies
☐	☒	Eukaryotic cell lines
☒	☐	Palaeontology
☒	☐	Animals and other organisms
☐	☒	Human research participants

Methods

n/a	Involved in the study	
☒	☐	ChIP-seq
☒	☐	Flow cytometry
☒	☐	MRI-based neuroimaging

Antibodies

Antibodies used	Abciximab (anti-GpIIb/IIIa, Reopro, Eli Lilly); anti-CD42b antibody (anti-GpIba receptor, HIP1 clone, Thermofisher); collagen type I antibody (AB755P, Millipore)
Validation	antibodies have been validated by the manufacture.

Eukaryotic cell lines

Policy information about cell lines

Cell line source(s)	Human Umbilical Vein Endothelial Cells (HUVEC) were purchased from Lonza
Authentication	Since cell line was purchased from reputable supplier. authentication was performed by the supplier.
Mycoplasma contamination	Since cell lines do not have sign of contamination, they were not tested for mycoplasma contamination.
Commonly misidentified lines (See ICLAC register)	No misidentified lines.

Human research participants

Policy information about studies involving human research participants

Population characteristics	Healthy volunteers of either sex between age 20 -30 yrs old.
Recruitment	Healthy adult volunteers of either sex were recruited following protocols approved by the UB Health Sci.ence Institutional Review Board (Buffalo, NY). Human blood was obtained by venipuncture from healthy adult volunteers.
